# Widespread intron retention and exon skipping characterise alternative splicing changes in a *C. elegans* model of spinal muscular atrophy

**DOI:** 10.1093/hmg/ddaf176

**Published:** 2025-12-01

**Authors:** Saman Rashid, Aykut Shen, Amy Yong, Alper Akay, Maria Dimitriadi

**Affiliations:** School of Health, Medicine and Life Sciences, University of Hertfordshire, College Lane, Hatfield, AL10 9AB, United Kingdom; School of Biological Sciences, University of East Anglia, Norwich Research Park, Norwich, NR4 7TJ, United Kingdom; School of Health, Medicine and Life Sciences, University of Hertfordshire, College Lane, Hatfield, AL10 9AB, United Kingdom; School of Biological Sciences, University of East Anglia, Norwich Research Park, Norwich, NR4 7TJ, United Kingdom; School of Health, Medicine and Life Sciences, University of Hertfordshire, College Lane, Hatfield, AL10 9AB, United Kingdom

**Keywords:** SMA, alternative splicing, intron retention, exon skipping, C. elegans

## Abstract

Spinal muscular atrophy (SMA) is a neurodegenerative disease caused by reduced levels of the survival motor neuron (SMN) protein, an essential component of the RNA splicing machinery. Although disruption of alternative splicing is a well-established hallmark of SMA, the specific splicing events that contribute to disease pathogenesis remain poorly understood. We utilised an established *Caenorhabditis elegans* SMA model to investigate global splicing changes using poly(A)+ RNA-seq and custom transcriptome assembly. Zygotic loss of *smn-1* led to extensive transcriptomic changes, including over 1000 alternative splicing events, many of which were functionally tied to larval development. Exon skipping and intron retention were the most prevalent splicing alterations, and sequence motif analysis indicated a general shift from strong to weak splice site usage; however, no single motif accounted for the majority of observed splicing changes. Notably, we identified an overlap between *smn-1* dependent splicing and those regulated by U6 snRNA m6A methylation. Our findings reinforce the conserved, broad role of SMN in maintaining splicing fidelity and reveal specific sequence biases associated with splicing errors in SMA.

## Graphical Abstract

**Graphical Abstract:**
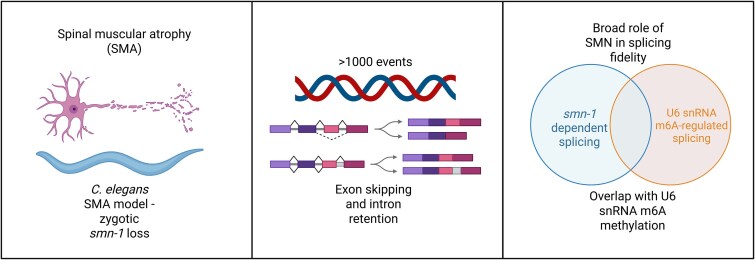

## Introduction

Spinal muscular atrophy (SMA) is an autosomal recessive motor neuron disorder characterised by the progressive degeneration of spinal α-motor neurons, leading to muscle dysfunction and atrophy [1]. In milder forms, the disease manifests as gradually worsening muscular weakness, whereas severe cases cause profound impairment of proximal neuromuscular function and are often fatal in early childhood [2]. SMA is characterised by a deficiency of the ubiquitously expressed survival motor neuron (SMN) protein, primarily due to mutation(s) or deletions in the *SMN1* gene [3, 4]. Although the closely related *SMN2* gene is present and capable of producing SMN protein, a single nucleotide polymorphism (C-to-T transition in exon 7) disrupts an exonic splicing enhancer, leading to exon 7 skipping and the production of a functionally impaired protein known as SMNΔ7 [3]. Due to a gene duplication event, patients may harbour multiple copies of *SMN2*, which contributes to the phenotypic variability observed in SMA. However, approximately 85–90% of *SMN2*-derived transcripts result in SMNΔ7, while only 10–15% produce full-length, functional SMN protein, which is insufficient to prevent the development of SMA [5].

The SMN protein is expressed in both the nucleus and cytoplasm, where its canonical function involves the assembly of small nuclear ribonucleoproteins (snRNPs), essential components of the pre-mRNA splicing machinery, through interactions with the Gemin complex [6–9]. Consequently, splicing defects represent a prominent feature of SMA pathology, with aberrant pre-mRNA splicing observed in various *in vitro* and *vivo* models [10–16]. Notably, in SMN-depleted NSC-34 cells, U snRNP biogenesis was significantly reduced, with exon skipping identified as the predominant splicing event following SMN depletion [17]. Interestingly, although U12-type introns have been proposed to be particularly sensitive to reduced SMN levels, Custer and colleagues [17] reported few instances of U12 intron retention in SMN-depleted cells; however, widespread U12 intron retention was observed across multiple tissues in SMA mouse models and patient fibroblasts [13, 14]. Similar findings were observed following transcriptomic analysis of SMA patient fibroblasts, in which a notable increase of U12-type intron retention was also observed [14]. Moreover, reductions in minor-class small nuclear RNAs (U12 snRNAs), accompanied by aberrant splicing of U12-type intron-containing transcripts, have been observed in both SMA patient-derived fibroblasts and SMA animal models. These findings further support a link between minor intron mis-splicing and SMA pathology [12, 18, 19]. Collectively, evidence from patient-derived cells and SMA vertebrate models demonstrates that SMN deficiency broadly impairs snRNP assembly, resulting in widespread splicing abnormalities—including exon skipping, intron retention, and altered splice site selection—in transcripts essential for neuronal function and survival [20, 21].

In the nematode *Caenorhabditis elegans* (*C. elegans*), *smn-1* is essential for viability, with complete loss of function resulting in severe developmental arrest, larval lethality, impaired neuromuscular activity and a dramatically shortened lifespan [22–24]. To maintain *smn-1(ok355)* null mutants, heterozygous parental strains are generated using the *hT2* balancer chromosome, which suppresses recombination and preserves the mutant allele in the population. Therefore, viable *smn-1* mutant progeny relies on maternally deposited SMN-1 protein from *smn-1(ok355)/hT2* heterozygous mothers, enabling embryonic development despite the absence of zygotic *smn-1* expression [22].

In *C. elegans*, depletion of *smn-1*—the ortholog of human *SMN1*—was noted to result in widespread changes in expression of mitochondrial, vacuolar H+-ATPase and histone genes [25]. Moreover, diminished *smn-1* levels caused reduced recognition of weak 3′ splice sites (SS), which was attributed to its interaction with the U2AF large subunit *uaf-1* [25, 26]. These observations were derived from comparison with wildtype animals at the early symptomatic stage. It is worth noting that unpaired DNA, such as that present in the *hT2* balancer chromosome, can influence histone methylation and gene expression [23, 27]. Therefore, utilising wildtype controls from a common *hT2* genetic background and analysing *smn-1(ok355)* nematodes at the symptomatic stage are essential to accurately assess the effects of *smn-1* depletion in *C. elegans*.

Despite significant advances in the field, the specific alternative splicing events or downstream gene targets that are most critical for disease progression following SMN depletion remain unresolved. This challenge is further compounded by the ubiquitous expression of SMN, which performs splicing-related functions across a diverse range of tissues beyond motor neurons, leading to widespread molecular perturbations. Whole-animal transcriptomic analyses provide an unbiased and comprehensive approach to delineate the global landscape of alternative splicing changes in response to SMN deficiency. Importantly, such studies have the potential to uncover novel splicing events and identify conserved molecular pathways disrupted in SMA, thereby offering new insights into disease mechanism(s) and potential therapeutic targets. In this study, we investigate the effects of zygotic *smn-1* loss in the presence of residual maternal SMN-1 protein on gene expression and splicing by profiling *smn-1(ok355)* mutants during the symptomatic phase, using wildtype controls derived from a shared *hT2* genetic background.

## Results

### Zygotic loss of *smn-1* leads to large-scale gene expression and splicing changes

The *smn-1(ok355)* null allele contains a large 975 bp deletion that leads to L4 larval stage arrest with defects in gonad development [22]. Therefore, the *smn-1(ok355)* strain is maintained in a heterozygous state using the *hT2* balancer chromosome (*smn-1(ok355)/hT2*). Although complete loss of SMN-1 is lethal, *C. elegans* homozygous for *smn-1* can survive for several days due to partial maternal rescue [22]. To investigate gene expression and splicing alterations resulting from *smn-1*, we performed paired-end Illumina sequencing of poly(A)+ RNA isolated from *smn-1(ok355)* progeny derived from heterozygous parents, alongside wildtype controls from mothers carrying the *hT2* balancer to maintain a consistent genetic background. Using three biological replicates, we obtained, on average, 24 million read pairs with a minimum of 98.2% mapped reads. To capture novel transcriptome isoforms and splicing events, we built a custom *C. elegans* reference transcriptome using the reads from *smn-1(ok355)* and wildtype animals and mapped reads to the custom transcriptome. Overall, the PCA analysis distinguished between the *smn-1(ok355)* and wildtype genotypes (Fig. S1A).

**Figure 1:**
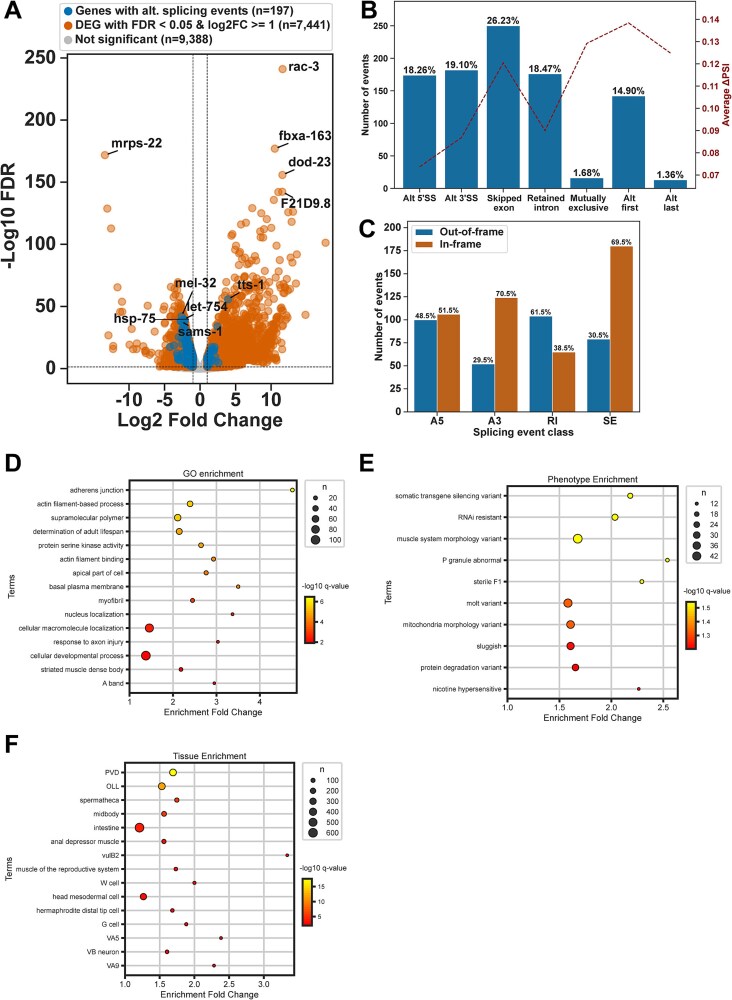
Zygotic loss of SMN-1 causes large-scale gene expression and splicing changes. (A) Volcano plot showing the log2 fold change of gene expression in *smn-1(ok355)* animals compared to wildtype animals. Genes that also show significant splicing changes are coloured in blue. (B) Number and percentage of significantly altered splicing event types (blue bars) and their average ΔPSI values in *smn-1(ok355)* animals compared to wildtype animals. (C) Number and percentage of in-frame and out-of-frame changes for alternative splicing classes, alternative 5′ (A5), 3′ (A3), retained intron (RI) and skipped exon (SE) in *smn-1(ok355)* animals compared to wildtype animals. (D) Gene ontology (GO) enrichment analysis of biological processes, cellular components and molecular functions were significantly enriched among the differentially spliced genes. (E) Phenotype enrichment analysis for the gene set, illustrating overrepresented phenotypic variants with enrichment fold changes. (F) Tissue-specific enrichment of genes impacted across various tissues, including neurons, muscles and reproductive system components.

Our differential gene expression analysis revealed large-scale gene expression changes with 4579 upregulated and 3059 downregulated genes in *smn-1(ok355)* animals compared to wildtype (Fig. 1A and Fig. S1B). We verified that the *smn-1* RNA levels are significantly reduced in *smn-1(ok355)* animals, showing only minimal *smn-1* reads likely arising from maternal contribution (Fig. S1C). Next, we compared the splicing differences using ΔPSI (per cent spliced in) analysis and identified 1023 differentially spliced genes across seven categories of splicing changes between *smn-1(ok355)* and wildtypes (Fig. 1B). Overall, 197 genes had significant changes in both their expression and splicing (Fig. 1A and Fig. S1D). The mean ΔPSI in each category of splicing defect indicated that exon skipping and alternative first and last exon usage events had the highest ΔPSI values (Fig. 1B). We did not observe any correlation between different splicing events and their expression (Fig. S1E and F).

To understand the impact of alternative splicing events on protein translation, we analysed whether the splicing changes affect the open reading frame of the transcripts (Fig. 1C). As expected, the majority of intron retention events led to out-of-frame changes, and most exon skipping events led to in-frame changes. The majority of 3′ splice site changes were in-frame, and alternative 5′ splice site events equally led to in and out-of-frame changes (Fig. 1C).

We conclude that the *smn-1* function is required for accurate splicing and expression of thousands of genes during the larval development of *C. elegans*, particularly impacting intron retention and exon skipping events.

### Functional and tissue enrichment analyses of *smn-1* targets

To further explore the biological relevance of *smn-1* affected genes, we performed comprehensive Gene Ontology (GO), phenotype and tissue enrichment analyses of differentially spliced genes. The GO analysis revealed significant enrichment in biological processes closely associated with larval development, including adherens junction organisation, actin filament-based processes, regulation of adult lifespan and muscle system morphology (Fig. 1D). Phenotype enrichment analysis highlighted key developmental and physiological traits such as somatic transgene silencing, RNAi resistance, muscle system morphology defects, P granule abnormalities, sterility and molting variants (Fig. 1E). These phenotypes highlight the involvement of *smn-1* affected genes in essential developmental, germline and early embryogenic functions. Tissue-specific enrichment pointed to a pronounced role for these targets in PVD neurons, the spermatheca, reproductive musculature and epidermal cells, all critical for proper growth and function during both larval and adult stages (Fig. 1F).

Collectively our data supports the hypothesis that a substantial subset of *smn-1* affected genes are functionally tied to larval development, somatic and germline tissues and post-embryonic processes integral to organismal growth and fertility [22, 28].

### Alternative 5′ splice site usage in *smn-1(ok355)* animals

**Figure 2:**
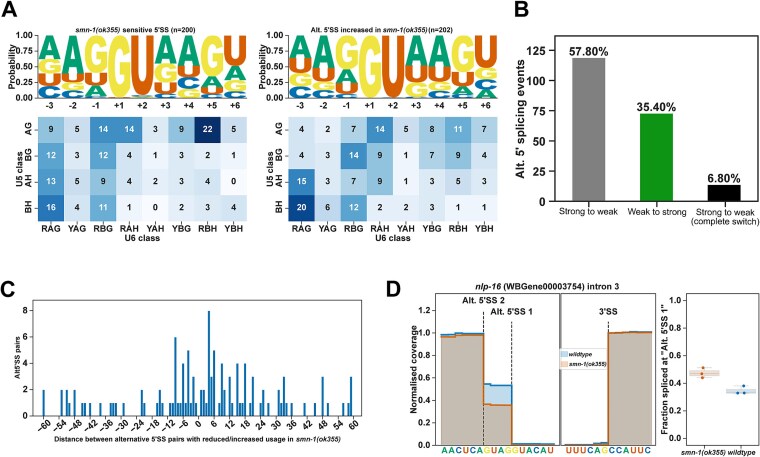
Zygotic loss of SMN-1 leads to alternative 5′ SS usage. (A) Sequence motif analysis of 5′ SSs sensitive to SMN-1 loss (left panel) and 5′SSs used more frequently in *smn-1(ok355)* animals. Frequency of different sequences for U5 interacting (y-axis) sequences (−2, −1 positions) and U6 interacting sequences (x-axis, +3, +4, +5 positions) are shown in the heat-map. (B) Alternative 5′SSs shifting from canonical (strong) to non-canonical (weak) positions (grey), from weak to strong positions (green) and those that completely shift from strong to weak positions (black) are shown. (C) Frequency of alternative 5′SS events and their distance to the original splice site position. (D*) nlp-16* intron 3 splice site coverage showing the wildtype (blue) and *smn-1(ok355)* (brown) splice site positions.

*C. elegans* 5′ splice sites predominantly have the //GURAGU motif (R = A or G, Fig. S2A). Mutations in splicing factors such as *snrp-27* and *snu-66* or the absence of U6 snRNA m6A modification can reduce the usage of 5′ SSs with //GURAG motif and promote the usage of 5′ SSs with AG//GU motif [29–31]. In contrast, the sequence motifs of 5′ SSs sensitive to loss of *smn-1* and 5′ SSs used more frequently in *smn-1(ok355)* are similar with only minor differences; *smn-1(ok355)* sensitive sites have more pronounced AG//GURAG motif and the alternative sites have less AG at the −3 and − 4 positions and less AG at the +4 and + 5 positions (Fig. 2A). The nature of the base at +3 or + 4 positions independently or together did not correlate with the ΔPSI changes in *smn-1(ok355)* animals (Fig. S2B-D). Together, these changes lead to a shift from stronger 5′ SSs used more frequently in wildtype animals to weaker 5′ SSs used less frequently in wildtype animals (Fig. 2B and E). Alternative 5′ SSs can be found upstream and downstream of the canonical splice site, most often within 20 nt (Fig. 2C). For example, *nlp-16* encodes for a nematode neuropeptide protein. In wildtype animals, intron 3 of *nlp-16* is equally spliced at two 5′ SSs (Fig. 2D). In *smn-1(ok355)* animals, the usage of alternative 5′ SS1 is reduced by approximately 15% (Fig. 2D).

In conclusion, zygotic loss of *smn-1* results in differential 5′ splice site usage; however, this altered splicing preference does not correspond to the +4A bias observed in other splicing pathway mutants affecting 5′ splice site recognition.

### Alternative 3′ splice site usage in *smn-1(ok355)* animals

**Figure 3:**
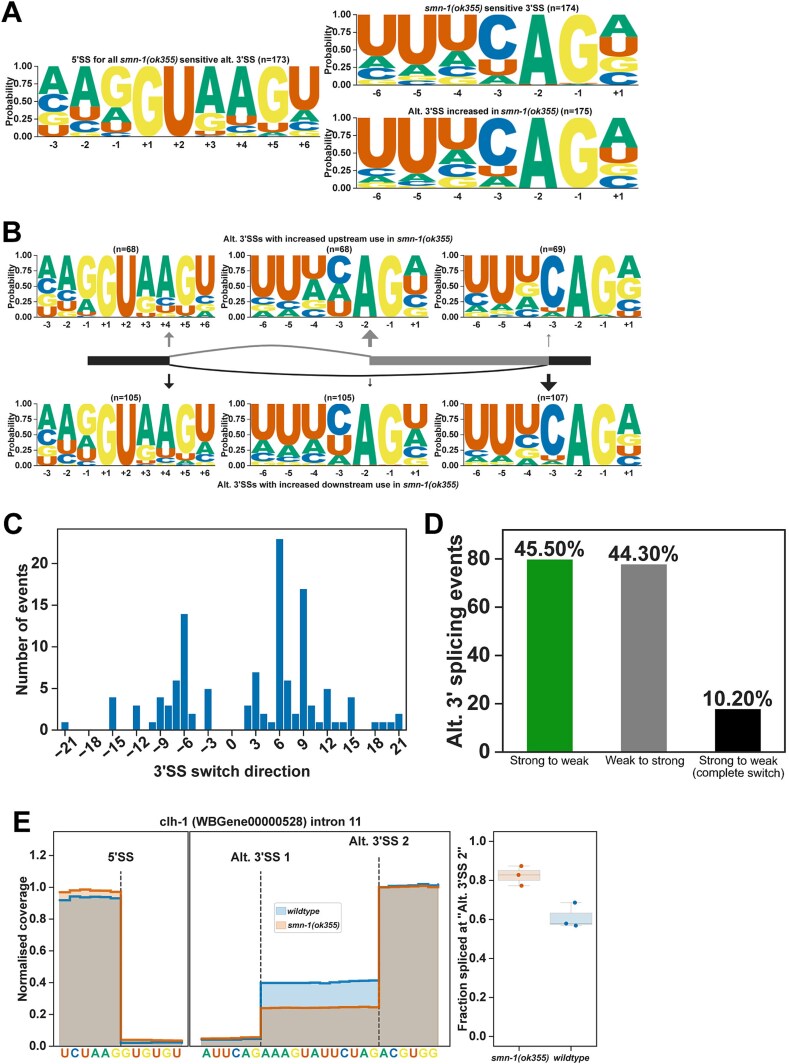
Zygotic loss of SMN-1 leads to alternative 3′ SS usage. (A) Sequence motifs of *smn-1* sensitive 3′SSs and 3′SSs used more frequently in *smn-1(ok355)*. (B) Sequence motifs of 3′SSs sensitive to loss of *smn-1* and those used more frequently separated by direction of splice site change. (C) Frequency of alternative 3′SS events and their distance to the original splice site position. (D) Alternative 3′SSs shifting from canonical (strong) to non-canonical (weak) positions (grey), from weak to strong positions (green), and those that completely shift from strong to weak positions (black) are shown. (E) *clh-1* intron 11 splice site coverage showing the wildtype (blue) and *smn-1(ok355)* (brown) splice site positions.

In *smn-1(ok355)* mutants, 19.1% of alternative splicing events are alternative 3′ SS usage (Fig. 1B). In order to understand if *smn-1* is required for the splicing of specific 3′ SS sequences, we analysed the sequence motifs around 3′ SSs. The sequence motif of 5′ SSs of all alternatively spliced 3′ SS events is similar to the general 5′ SS sequences with the AG//GURAG motif (Fig. 3A). The sequence motif of *smn-1* sensitive 3′ SSs and the 3′ SSs used more often in *smn-1(ok355)* mutants are highly similar and reminiscent to the canonical *C. elegans* 3′ SS motif of UUUCAG/R motif with minor differences; the *smn-1* sensitive sites have less frequent -3C and both sensitive and alternative sites have a preference for +1A/U instead of +1A/G (Fig. 3A). In contrast, alternative 3′ SSs with increased upstream or downstream usage show differences in their sequence motifs; alternative 3′ SSs with increased upstream usage appear to shift from a stronger downstream position to a weaker upstream usage, and 3′ SSs with increased downstream position appear to shift from a weaker upstream position to a stronger downstream position (Fig. 3B) and there is no correlation with the 5′ SS +4A (Fig. S3A). Overall, most alternative 3′ SSs are downstream of the canonical 3′ SSs (Fig. 3C), but the shift from weak to strong or strong to weak is approximately equal (Fig. 3B and D). Similar to other splicing mutants in *C. elegans*, 3′ SSs shifts in *smn-1(ok355)* show 3 nt periodicity starting from −6 or + 6 positions. For example, *clh-1* intron 11 has 2 alternative splicing positions, and the usage of the upstream alternative 3′ SS 1 position is reduced from approximately 40% in wildtype animals to 20% in *smn-1(ok355)* (Fig. 3E).

Overall, the 3′ SS changes in *smn-1(ok355)* animals do not correlate with the 3′ SS strength determined by the U2AF binding preferences.

### Intron retention and exon-skipping in *smn-1(ok355)* animals

**Figure 4:**
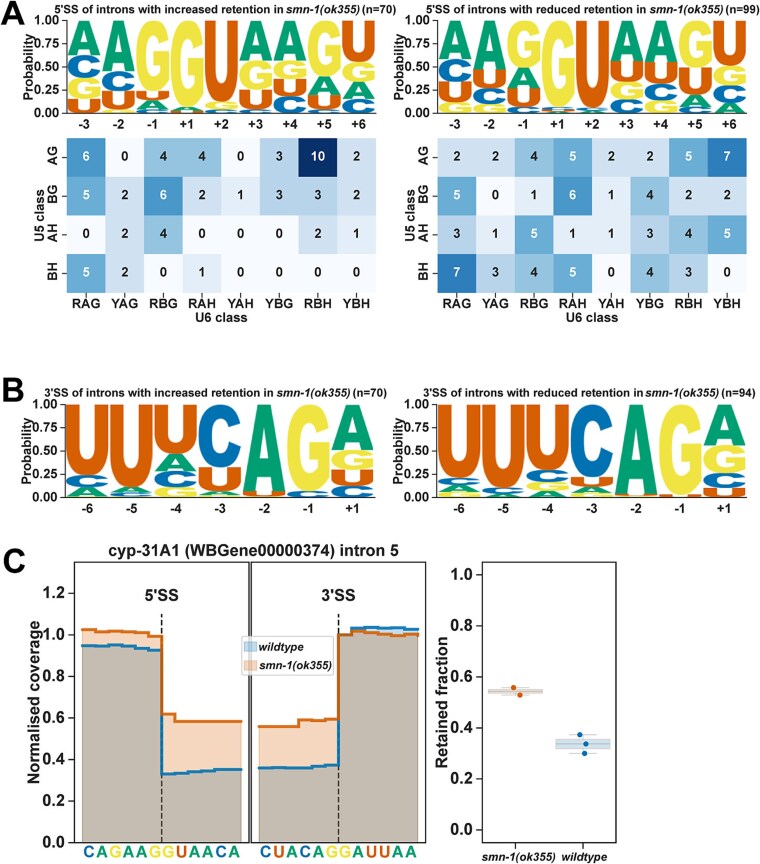
Zygotic loss of SMN-1 leads to changes in intron retention. (A) 5′ SS motifs of introns with increased retention (left panel) or reduced retention (right panel) and the frequency of bases that interact with U6 and U5 snRNAs (heatmap). (B) 3′ SS motifs of introns with increased retention (left panel) or reduced retention (right panel). (C) *cyp-31A1* intron 5 coverage map showing increased retention in *smn-1(ok355)*.

**Figure 5:**
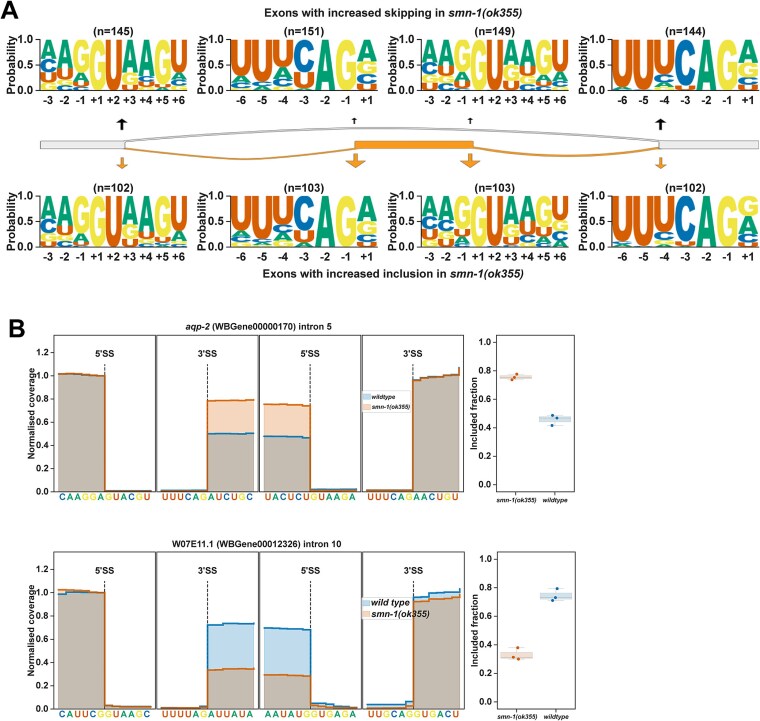
Zygotic loss of SMN-1 leads to exon-skipping and exon-inclusion. (A) 5′ and 3′ SS motifs of exons with increased skipping (upper panel) and increased inclusion (lower panel) together with the upstream 5′ SS and downstream 3′ SS sequence motifs. (B) *aqp-2* intron 5 with increased inclusion in *smn-1(ok355)* animals compared to wildtype animals. *W07E11.1* intron 10 shows increased skipping in *smn-1(ok355)* animals compared to wildtype animals.

In *smn-1(ok355)* animals, intron retention and exon-skipping events have the highest ΔPSI values. Therefore, we analysed the sequence motifs of 5′ and 3′ SSs of the intron retention and exon-skipping events (Fig. 4 and 5). The 5′ SSs of introns with increased retention in *smn-1(ok355)* animals (n = 70) have predominantly an AG//GU motif and lack +4A required for U6 snRNA m6A base pairing (Fig. 4A). However, the 5′ SSs of introns with reduced retention in *smn-1(ok355)* animals (n = 99) have reduced AG motif at −1 and − 2 positions, and + 4A frequency is slightly higher (Fig. 4A). Similarly, the 3′ SS motifs of introns with increased or decreased retention are similar and consist mainly of UUUCAG/R sequence, with the introns showing increased retention having less frequent -4 U and -3C (Fig. 4B). For example, *cyp-31A1* intron 5 has a 5′ SS of AG//GUAAC, and 3′ SS of CUACAG//G, which are different to the canonical 5′ and 3′ SS motifs. As a result, *cyp-31A1* intron 5 shows significantly higher retention levels in *smn-1(ok355)* animals (Fig. 4C, additional examples Fig. S4).

In *smn-1(ok355)* animals, the highest number of splicing changes with the strongest ΔPSI values are the exon skipping events (Fig. 1B). The 5′ SSs of exons with increased skipping (Fig. 5A, top panels) or inclusion (Fig. 5A, bottom panels) have weaker splice site motifs compared to their corresponding upstream 5′ SSs, with less frequent AG at positions −2 and − 1 and less frequent AG at positions +4 and + 5. However, the 5′ SS motifs are similar between exons showing increased skipping or inclusion. The 3′ SSs of exons showing increased skipping or inclusion have similar motifs and are weaker than their downstream 3′ SSs with less frequent UC at positions −4 and − 3. Overall, although exon skipping or inclusion appears to associate with weaker 5′ and 3′ SS motifs, the sequence motifs alone cannot distinguish skipping events from inclusion events. For example, the *C. elegans* aquaporin gene *aqp-2* intron 5 inclusion is significantly reduced, whereas the dihydropyrimidine dehydrogenase orthologue *W07E11.1* intron 10 inclusion is significantly increased in *smn-1(ok355)* animals (Fig. 5B, additional examples Fig. S5A and B).

Overall, zygotic loss of *smn-1* causes large-scale changes in intron retention and exon-skipping or inclusion.

### Overlap of *smn-1* dependent splicing changes with other splicing factors

**Figure 6:**
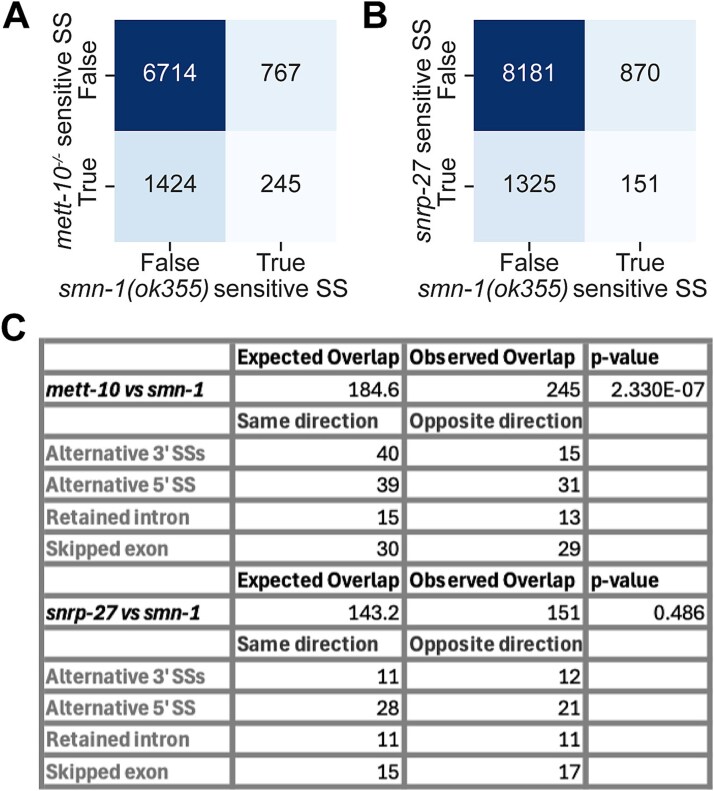
Overlap of SMN-1 sensitive splice sites with those affected from the loss of splicing factors. (A) Heatmap showing the overlap of splice sites sensitive to the loss of SMN-1 and splice sites sensitive to the loss of METT-10. (B) Heatmap showing the overlap of splice sites sensitive to the loss of SMN-1 and splice sites sensitive to the loss of SNRP-27. (C) Table summarising the splice site overlaps.

Mutations in spliceosome components that are directly involved in splice site recognition lead to splicing defects in a sequence-dependent manner. For instance, the absence of U6 snRNA m6A modification in yeast, *C. elegans*, plants, and humans, along with the absence of the splicing factors likely involved in 5′ SS recognition, such as *snrp-27* in *C. elegans*, predominantly affects 5′ SSs with +4A [29, 30, 32, 33]. The lack of a specific motif in splice sites affected by the zygotic loss of *smn-1* suggests that SMN-1 is not directly involved in splice site recognition. To investigate whether SMN-1 regulates the same splice sites as U6 snRNA m6A modification or *snrp-27*, we compared splice site alterations resulting from *smn-1* loss with those observed following the loss of the U6 snRNA m6A methyltransferase *mett-10* [30] and the spliceosome component *snrp-27* [29]. We identified significant overlap between *smn-1* and *mett-10* sensitive splice sites (Fig. 6A and C). Next, we compared whether the splicing changes were affected in the same direction between conditions (Fig. 6C). Approximately an equal number of splice sites were affected in the same direction as in the opposite direction, except for alternative 3′ SSs, where we observed that the majority of changes were affected in the same direction. In contrast, we did not see a significant overlap between *smn-1* affected splice sites and those affected in *snrp-27* (Fig. 6B). Our results suggest that *smn-1* may modulate splice site selection together with U6 snRNA m6A modification, implicating a potential mechanistic link between SMN-1 function and RNA methylation-dependent splicing regulation.

## Discussion

To date, identifying a definitive pathological mechanism directly linking SMN deficiency to neurodegeneration has remained elusive. In SMA models, no single molecular pathway has been identified whose correction universally mitigates the effects of SMN deficiency across diverse genetic backgrounds. This is expected, as SMN is essential for spliceosome assembly and thus impacts numerous cellular processes. This absence of a unifying pathogenic mechanism has been highlighted in multiple studies, underscoring the multifactorial nature of SMA and the insufficiency of targeting a single pathway to fully rescue the phenotype [34, 35].

To assess the effect of zygotic SMN loss during the symptomatic stage, we performed whole-animal transcriptomic profiling in *C. elegans*. Our analysis revealed that *smn-1(ok355)* mutants, which lack zygotic SMN-1 protein but retain maternally deposited SMN-1, exhibited widespread changes in gene expression and alternative splicing, underscoring the critical role of SMN-1 in transcriptome regulation during larval development (Figs 1A–C, S1A–F). These findings mirror prior observations from vertebrate SMA models, where SMN deficiency leads to defects in snRNP biogenesis and splicing regulation [14, 20]. Our findings that SMN depletion perturbs splicing and potentially other aspects of RNA processing are consistent with previous studies showing that SMN deficiency leads to widespread defects in RNA maturation [15, 17]. Notably, this includes impaired U7 snRNP-mediated histone RNA processing [36], highlighting the broader impact of SMN disruption on multiple RNA regulatory pathways.

Moreover, our observation that widespread alternative splicing alterations occur in *C. elegans smn-1(ok355)* mutants at the stage of developmental arrest is consistent with findings in vertebrate models, where extensive splicing disruptions coincide with symptom onset and precede overt neurodegeneration in SMA [12]. Our enrichment analyses provide key insights into the biological roles of *smn-1* sensitive splicing targets, highlighting their importance not only in neuromuscular function but also in broader developmental and reproductive processes. The significant enrichment for processes such as adherens junction organisation, actin cytoskeleton dynamics and muscle morphology (Fig. 1D) aligns with the essential role of SMN-1 in maintaining neuromuscular integrity and organismal motility, as observed in both *C. elegans* and vertebrate SMA models [15, 20, 22]. Phenotype enrichment implicating RNAi resistance and somatic gene silencing (Fig. 1E) reflects the known interplay between RNA regulatory pathways and developmental gene expression programs, suggesting that disruption of epigenetic and post-transcriptional control may contribute to the pleiotropic effects of SMN deficiency [14, 23]. In addition, tissue-specific enrichment in PVD neurons, reproductive musculature and epidermal cells (Fig. 1F) corroborates previous findings illustrating the systemic consequences of *smn-1* depletion across somatic and germline tissues during *C. elegans* larval development and adulthood [22, 25]. This emphasises that SMA pathology likely stems from complex, tissue-wide perturbations in splicing and gene expression rather than motor neuron dysfunction exclusively. Our data therefore extends our current understanding of SMN function, proposing that post-embryonic processes fundamental to growth and fertility are also vulnerable to splicing defects stemming from *smn-1* depletion, potentially underlying the developmental arrest phenotypes observed in *smn-1(ok355)* mutants.

Among the splicing changes, exon skipping and alternative usage of first and last exons exhibited the largest ΔPSI values (Fig. 1B). Intron retention events were also prominent, often involving introns with weak or non-canonical splice sites, such as in *cyp-31A1* (Fig. 4C). These results suggest that SMN-1 is particularly important for the accurate processing of transcripts with suboptimal splice signals. Interestingly, many retained introns and skipped exons lacked distinct sequence features that could reliably predict their mis-splicing, suggesting that SMN-1 influences splicing primarily by modulating overall spliceosomal efficiency rather than through recognition of specific sequence motifs. This agrees with studies in both vertebrate and invertebrate systems, which have established that SMN is indispensable for snRNP biogenesis and consequent pre-mRNA splicing [6, 8]. Widespread, tissue-specific reductions in snRNP abundance, along with disruption of both major and minor spliceosome components, have been reported in human SMA patient samples and animal models. These findings support a unifying model in which global splicing dysfunction represents a central pathogenic mechanism in SMA [18, 21].

Notably, our analysis of 5′ splice site usage revealed a bias toward weaker splice sites in *smn-1(ok355)* animals (Fig. 2B), while 3′ splice site usage revealed a shift from a weaker upstream position to a stronger downstream position, albeit with no particular favour in either direction (Fig. 3D). For 5′ SSs, we observed a shift from stronger canonical motifs to weaker, non-canonical sites, but this did not correspond to the +4A sequence preference seen in other splicing mutants such as *snrp-27* or *mett-10* (Fig. S2B–D). Similarly, 3′ SS usage often shifted downstream but without a clear pattern in splice site strength based on U2AF recognition motifs (Fig. 3A–D). This suggests that SMN-1 does not directly regulate splice site recognition specificity but may instead affect spliceosome assembly or fidelity more broadly. Indeed, it has been shown previously that the *C. elegans uaf-1* gene, which encodes the U2AF large subunit, could affect the behaviour and lifespan of *smn-1(ok355)* animals [25]. The discrepancy observed between our findings and those of Gao et al. [25] regarding U2AF and *smn-1* likely stems from differences in the developmental stage at which the animals were examined. While our analysis focused on symptomatic animals at the final larval stage, Gao and colleagues [25] assessed animals at an earlier symptomatic stage. Additionally, this comparison was made using wildtype animals that were not derived from the *hT2* genetic background. Nevertheless, the preferential retention of introns with weak or non-canonical splice sites is consistent with observations in SMN-deficient mouse tissues and human patient lymphoblasts, suggesting conserved vulnerability of such transcripts across species [11, 14].

We also compared splicing events affected by *smn-1* loss to those in *mett-10* and *snrp-27* mutants. A significant overlap was observed between *smn-1* and *mett-10*, particularly for 3′ SS events, whereas no significant overlap was found with *snrp-27* (Fig. 6A–C). This supports the model proposed by Lotti and coauthors [20], in which SMN deficiency leads to a general decrease in splicing efficiency rather than selective misrecognition of specific splice sites. Indeed, SNRP-27 promotes distal weak 3' SS usage, whereas U6 snRNA m6A modification promotes proximal weak 3' SS usage [30]. Importantly, although splicing changes in *smn-1* mutants partially overlap with those observed upon U6 snRNA m6A methylation, we found that *smn-1* sensitive 5′ splice sites do not exhibit the characteristic +4A to +4 U motif shift. This suggests that the impact of SMN-1 on alternative splicing is not simply mediated through U6 snRNA m6A-dependent 5’SS selection but may reflect a broader or position-dependent effect on spliceosome assembly or activity. Thus, while RNA methylation–dependent regulation of splice site choice is a general mechanism, its influence in the context of SMN deficiency appears to be more subtle and is unlikely to account for the majority of splicing alterations observed here. Therefore, the interaction between SMN-1 and U6 snRNA m6A modification could be splice site dependant and SMN-1 function in snRNP biogenesis could contribute to position dependent splicing changes. Consistent with this, experimental evidence from human, *Drosophila* and *C. elegans* suggests that SMN-1 is involved in the Sm class snRNAs U1, U2, U4 and U5 stability but not the Sm class U6, implying that the splicing changes and overlaps with U6 could be due to alterations in Sm class snRNAs [26, 37].

Altogether, our results demonstrate that the zygotic loss of SMN-1 in *C. elegans* causes widespread disruption of pre-mRNA splicing and gene expression. The patterns of intron retention and exon skipping, along with the shifts in splice site usage, point to a global reduction in splicing efficiency rather than misrecognition of specific splice site sequences. Moreover, our findings suggest that therapies aimed at broadly enhancing spliceosome function or stabilising weak splice sites may complement SMN-targeted approaches in SMA*.* These findings underscore the pivotal role of SMN in maintaining transcriptome integrity and establish a foundational dataset for future investigations aimed at delineating which splicing defects contribute most directly to SMA pathogenesis.

## Materials and methods

### *C. Elegans* strains and maintenance

LM99 [*smn-1(ok355)/hT2(I;III)*] and HA1981 [*+/hT2(I;III)*] were maintained at 20°C under standard conditions [38]. HA1981 was used as the wildtype control strain to maintain a common genetic background derived from mothers harbouring the *hT2* balancer.

### RNA extraction and sequencing

Total RNA was isolated from synchronised populations of *C. elegans* using TRIzol reagent, following standard protocols. Briefly, animals were washed and pelleted by centrifugation, then lysed in TRIzol (ThermoFisher). Samples were subjected to repeated freeze–thaw cycles in liquid nitrogen and a 37°C heating block to ensure complete lysis, followed by phase separation with chloroform (ThermoFisher). The aqueous phase was collected, and RNA was precipitated with isopropanol (ThermoFisher), washed with 75% ethanol, and air-dried before resuspension in nuclease-free water. RNA concentration and purity were determined spectrophotometrically. RNA integrity was assessed by electrophoresis on a 1% agarose-TBE bleach gel, and samples displaying clear 28S, 18S, and 5S rRNA bands with a 28S:18S ratio of ~ 2:1 were considered high quality. RNA sequencing was performed by Novogene. Specifically, mRNA was purified using poly-T oligo-attached magnetic beads, fragmented, and used for first- and second-strand cDNA synthesis. After end repair, adaptor ligation, and size selection (150–200 bp), PCR amplification was performed and library quality was verified on the Agilent Bioanalyzer. Libraries were sequenced on an Illumina platform to generate paired end reads. Raw reads in FASTQ format were filtered with fastp to remove reads containing adapters, poly-N sequences, or low-quality bases. Data quality metrics (Q20, Q30, GC content) were calculated for the clean reads, which were used for all downstream analyses.

### RNA-seq read preprocessing and quality assessment

Standard adapter sequences and common contaminants were removed from the 150 bp non-stranded paired-end reads using BBduk from the BBtools package [39] (version 37.62). Bases with a Phred quality score below 15 were trimmed and only reads longer than 34 bases were retained. Read quality was assessed using FastQC (https://www.bioinformatics.babraham.ac.uk/projects/fastqc/) (version 0.12.1) and summarised with MultiQC [40] (version 1.13). Reads were aligned to the *C. elegans* WBcel235 reference genome using STAR [41] (version 2.7.8a) using the parameters described by Shen et al. [30]. A splice junction database was generated from the Ensembl release 95 reference annotation. Strand orientation of spliced reads was inferred from intron motifs.

### Transcriptome assembly, quantification and alternative splicing

Transcriptome assembly and downstream analyses followed the approach described by Parker et al. [33] and Shen et al. [30], where applicable, using the parameter settings optimised for *C. elegans* as described in the latter. For full methodological details, refer to the cited studies. In brief, condition-specific transcriptomes were *de novo* assembled using StringTie [42] (version 2.1.7) from pooled Illumina RNA-seq alignments in non-stranded mode. A unified transcript set was constructed by cross-referencing these assemblies with the reference annotation. Transcripts were quantified using Salmon [43] (version 1.10.1), with the *C. elegans* reference genome employed as a decoy. Alternative splicing events were annotated, and event-level inclusion (PSI) values were estimated using SUPPA2 [44] (version 2.3) with a minimum total expression of 10 per event. To evaluate the association between genotype and PSI values, generalised linear models were fitted per splicing event using Python’s statsmodels library [45–47] (version 0.11). P-values were adjusted for multiple testing using the Benjamini–Hochberg false discovery rate (FDR) method. Splicing events with significant PSI changes between wildtype and the *smn-1(ok355*) strains were identified using an FDR threshold of 0.05. Sequence logos were generated using matplotlib_logo (https://github.com/ mparker2/matplotlib_logo) and significant motif differences were identified with a likelihood-ratio test (G-test). Contingency tables for splice site classes at U5 and U6 interacting positions were created based on sequence deviations at the −2 to −1 positions from the AG consensus and the +3 to +5 positions from the RAG motif at the 5′ splice site, respectively. Gene tracks were constructed and visualised using pyBigWig [48] (version 0.3.18), pysam [49–51] (version 0.21.0), and matplotlib [52] (version 3.7.1).

### Differential expression analysis between wildtype and *smn-1(ok355)* strains

The abundance of transcripts was estimated using Salmon [43] version 0.11.2 in non-stranded mode. Transcript abundances were aggregated to the gene level using tximport [53]. Differential gene expression analysis, comparing *smn-1(ok355)* and wildtype strains, was performed in R [54] version 3.5, using edgeR [55] version 3.22.5. Genes showing differences in relative expression were identified using a false discovery rate (FDR) threshold of 0.05 and a log2 fold change of 1. The resulting gene set was used to explore the overlap between alternatively spliced and differentially expressed genes (see Fig. 1A). This overlap was also examined by cross-referencing the significantly alternatively spliced genes with those that were differentially expressed, regardless of the magnitude of the expression change. In that, a gene was considered differentially expressed if its expression-level FDR value met the 0.05 threshold (see Fig. S1D–E).

### Enrichment analyses for alternatively spliced genes

Phenotype, tissue, and gene ontology enrichment analyses were conducted using the WormBase Enrichment Suite [56, 57] focusing on the genes exhibiting significant (*p* < 0.05) splicing differences between *smn-1(ok355)* and wildtype animals. All *C. elegans* genes with annotated terms were used as background. A q-value of 0.1 was set as the significance threshold for identifying enriched terms.

### Comparative analysis of splicing changes across *mett-10*, *snrp-27*, and *smn-1* mutants

Illumina RNA-seq datasets for the wildtype N2 Bristol and ALP010 *mett-10(ok2204)III* derived from backcrossing of VC1743 (referred to as *mett-10−/−*) strains, and the CB936 *unc-73(e936)I* and SZ118 *unc-73(e936)I;snrp-27(az26)* (referred to as SNRP27 [M141T]) strains, were obtained from the European Nucleotide Archive (ENA) and Gene Expression Omnibus (GEO), under accession numbers PRJEB65287 [30] and GSE113275 [29], respectively. The *mett-10−/−* dataset was processed following the method described by Shen et al. [30] to create a *de novo* transcriptome that includes splicing changes specific to *mett-10−/−*. These transcripts were then quantified using Salmon for the CB936 *unc-73(e936)I* and SZ118 *unc-73(e936)I;snrp-27(az26)*I strains as described in the original study [30]. Transcript quantification was also carried out for wildtype and *smn-1(ok355)* strains using the same custom transcriptome and following the method described earlier in this study. Splicing differences relative to control datasets were identified in SZ118 *unc-73(e936)I;snrp-27(az26)*I as described by Shen et al. [30], and in *smn-1(ok355)* animals using the method described earlier. Events detected in *smn-1(ok355)* were then cross-referenced with those detected in SZ118 *unc-73(e936)I;snrp-27(az26)*I. *C. elegans* and those already reported for *mett-10−/−* in the same study. The likelihood of concurrent splice site usage across conditions was assessed using Pearson’s chi-square test of independence.

## Supplementary Material

Supplementary_Figures_ddaf176

## Data Availability

All raw and experimental data have been deposited in ArrayExpress (https://www.ebi.ac.uk/biostudies/arrayexpress) under accession E-MTAB-16084.
