# Supplementary material for: Widespread intron retention and exon skipping characterise alternative splicing changes in a C. elegans model of spinal muscular atrophy
Source: Hum Mol Genet. 2025 Dec 1;35(2):ddaf176. doi: 10.1093/hmg/ddaf176 (PMC13158239; doi:10.1093/hmg/ddaf176)
Supplement: Supplementary_Figures_ddaf176 [file supplementary_figures_ddaf176.pdf]

Figure S1

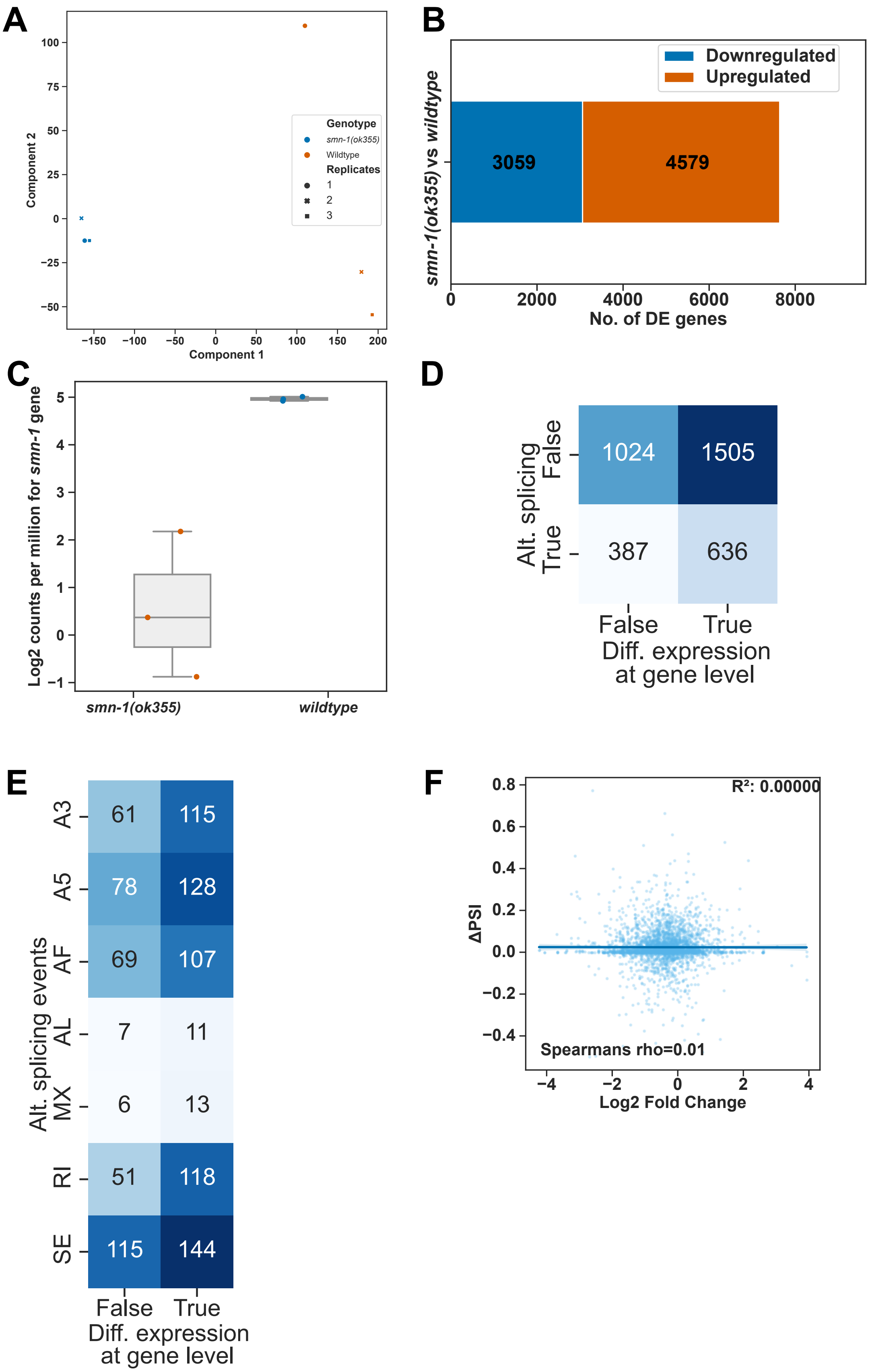

Figure S1. Principal component, differential expression, and alternative splicing analysis reveals widespread transcriptomic dysregulation in *smn-1(ok355)* mutants. (A) Principal component analysis of sequencing data and replicates. (B) Up and downregulated gene totals in *smn-1(ok355)* animals compared to wildtype animals. (C) Read counts mapping to *smn-1* gene in *smn-1(ok355)* animals compared to wildtype animals. (D) Heatmap showing the overlap of genes with significant alternative splicing and genes with significant differential expression. (E) Heatmap showing the overlap of differential expressed genes and the subclass of splicing events (SE-skipped exon, RI-retained intron, MX-mixed, AL-alternative last exon, AF-alternative first exon, A5-alternative 5′ SS and A3-alternative 3′ SS. (F) Spearmans correlation of PSI values of genes with alternative splicing and log2 fold change of genes with differential expression.

Figure S2

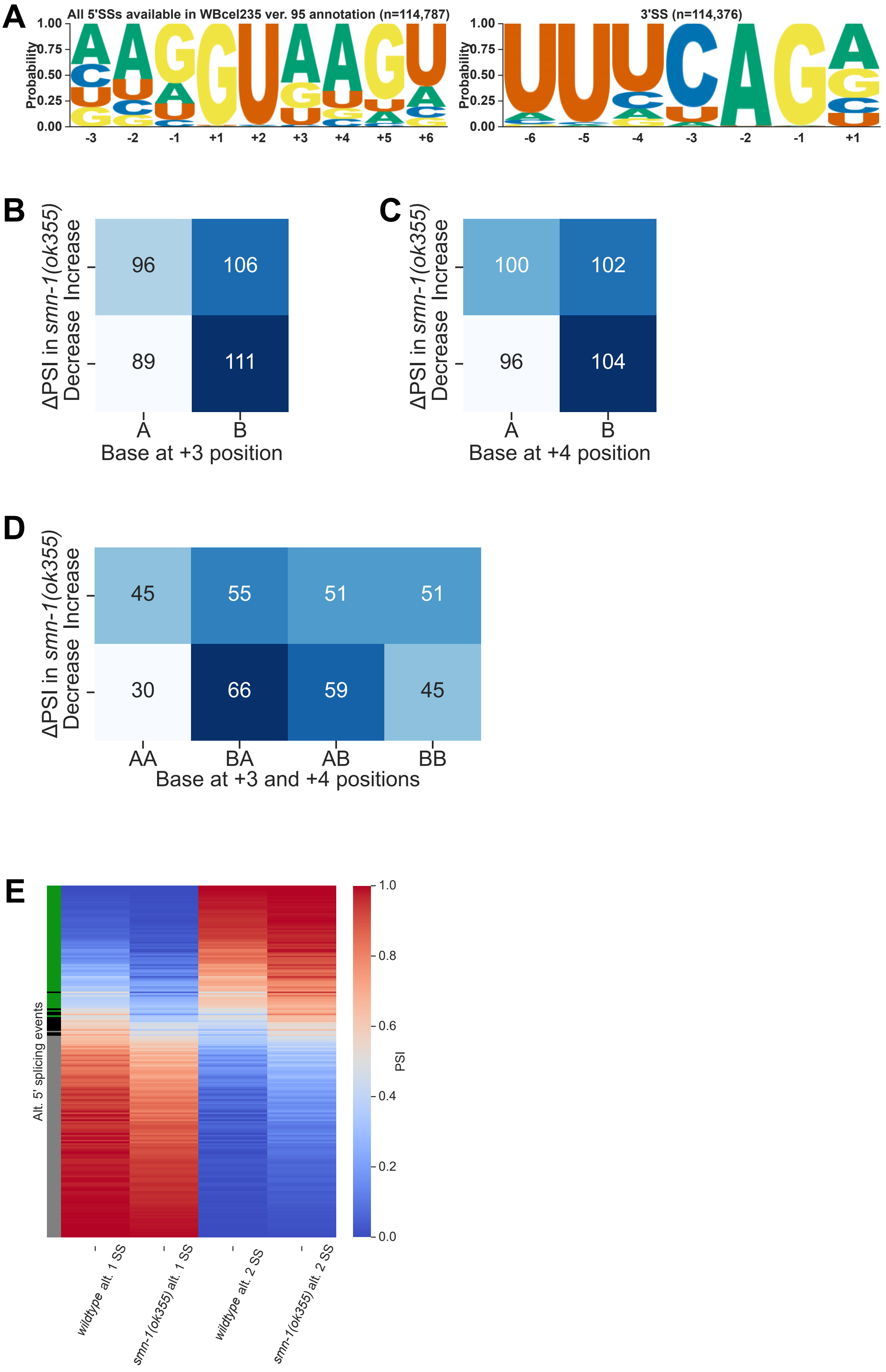

Figure S2. Sequence motifs and positional effects at 5′ splice sites associates with splicing alterations in *smn-1(ok355)* mutants. (A) Sequence motifs all 5′ and 3′ SSs in WBcel235 ver. 95 genome release. (B) Heatmap showing the direction of ΔPSI change and the presence of "A" or other bases at position +3 of 5′ SSs. (C) Heatmap showing the direction of ΔPSI change and the presence of "A" or other bases at position +4 of 5′ SSs. (D) Heatmap showing the direction of ΔPSI change and the base positions at +3 and +4 positions of 5′ SSs. (E) 5′ SS ΔPSI change between splice site positions in *smn-1(ok355)* animals compared to wildtype animals.

Figure S3

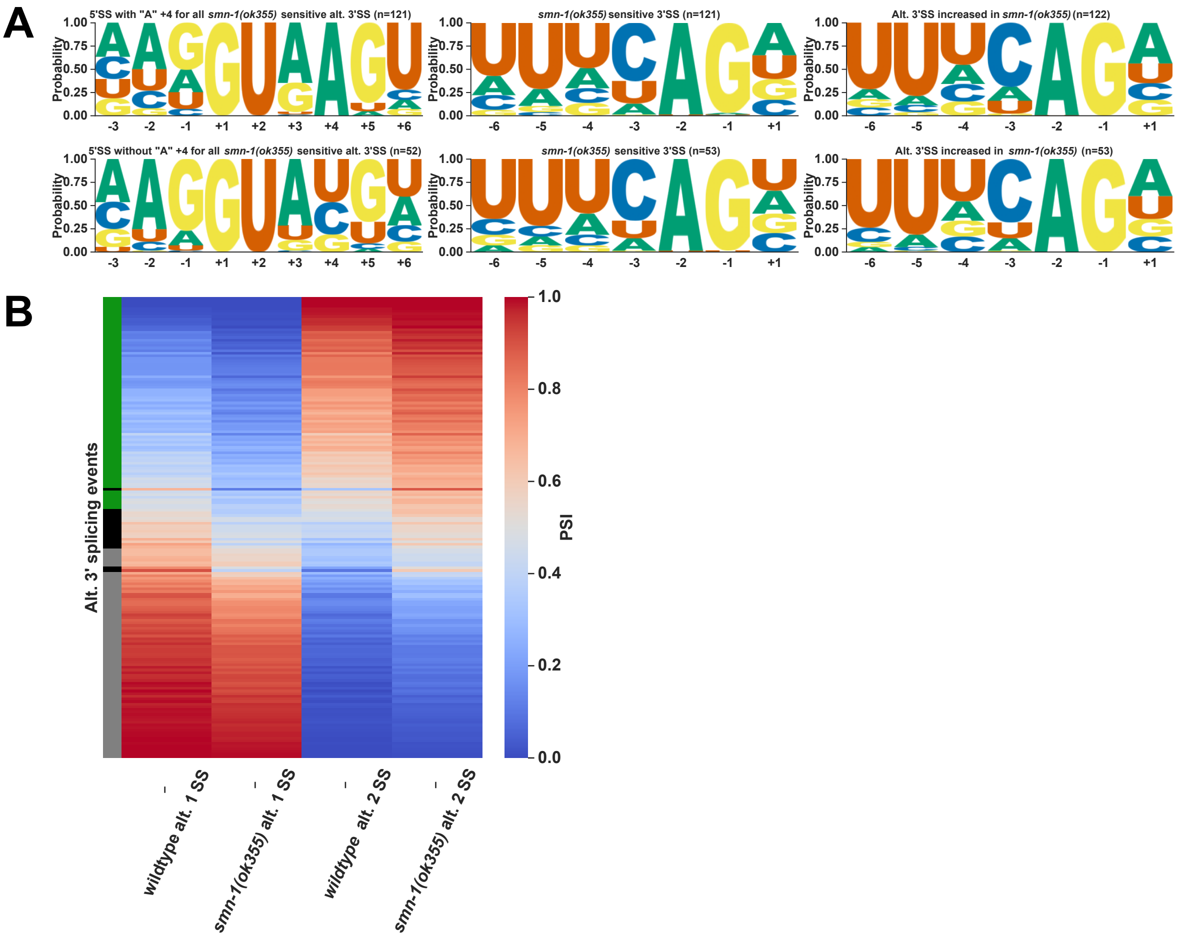

Figure S3. Sequence features and positional dynamics of alternative 3′ splice site usage in *smn-1(ok355)* mutants. (A) Sequence motif analysis of alternative 3′ SS positions separated with respect to +4A presence at the upstream 5′ SS. (B) 3′ SS ΔPSI change between splice site positions in *smn-1(ok355)* animals compared to wildtype animals.

Figure S4

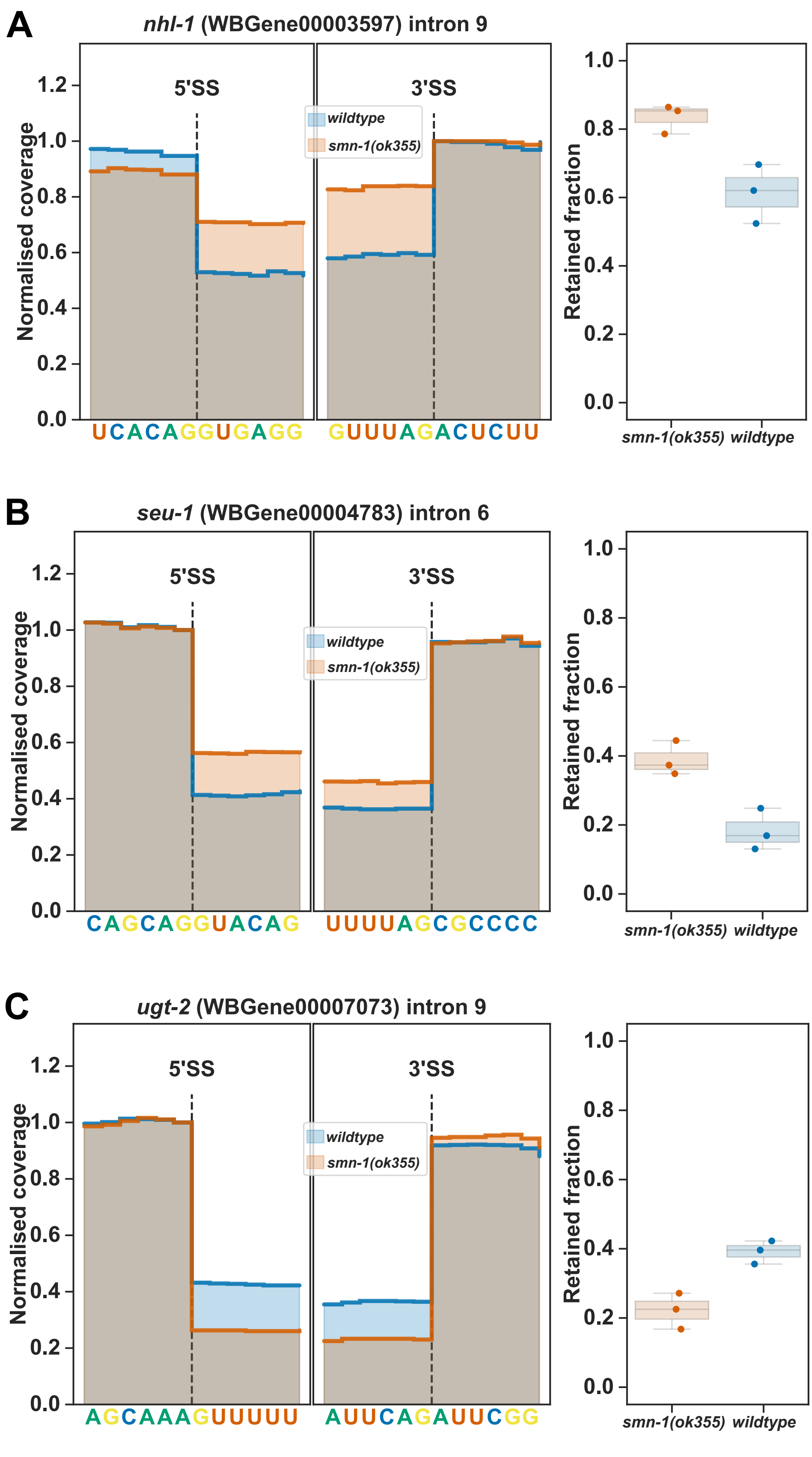

Figure S4. Additional examples of intron retention events in specific transcripts. (A) *nhl-1* intron 9 coverage map showing increased retention in *smn-1(ok355)*. (B) *seu-1* intron 6 coverage map showing increased retention in *smn-1(ok355)*. (C) *ugt-2* intron 9 coverage map showing reduced retention in *smn-1(ok355)*.

Figure S5

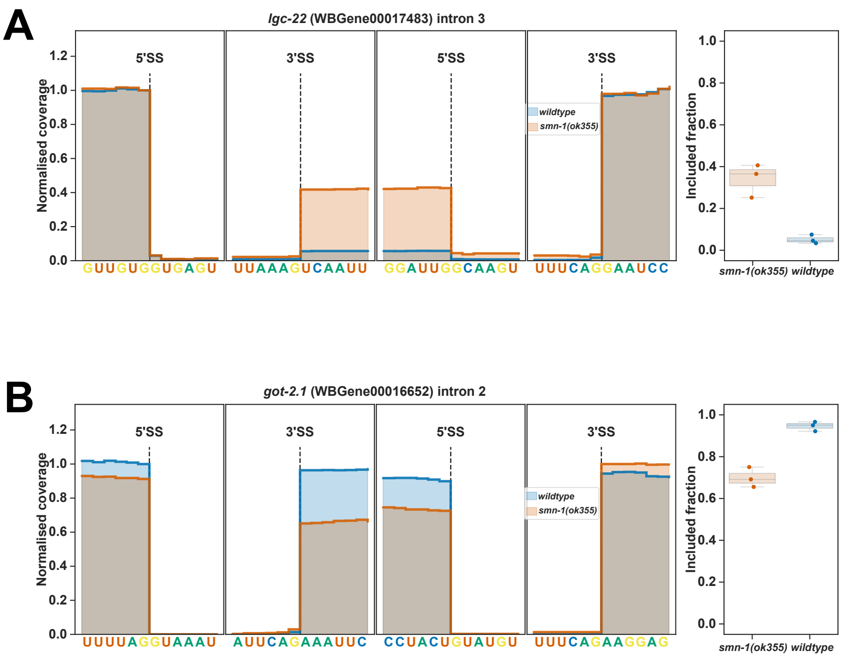

Figure S5. Additional examples of exon skipping or inclusion events in specific transcripts. (A) *lgc-22* intron 3 with increased inclusion in *smn-1(ok355)* mutants compared to wildtype controls. (B) *got-2.1* intron 2 with increased skipping in *smn-1(ok355)* mutants compared to wildtype controls.
